# Antibody Surface Profiling Identifies Glycoforms in Multiple Myeloma as Targets for Immunotherapy: From Antibody Derivatives to Mimetic Peptides for Killing Tumor Cells

**DOI:** 10.3390/cancers15071934

**Published:** 2023-03-23

**Authors:** Mouldy Sioud, Anniken Olberg

**Affiliations:** Department of Cancer Immunology, Division of Cancer Medicine, Oslo University Hospital-Radiumhospitalet, Ullernchausseen 70, 0379 Oslo, Norway

**Keywords:** multiple myeloma, targeted therapies, antibodies, lytic peptides, heparan sulfate

## Abstract

**Simple Summary:**

Today, many incurable blood malignancies, such as multiple myeloma (MM), acute myeloid leukemia, and myelodysplastic syndrome, lack effective immunotherapy options. In this study, we used phage display technology to develop candidate therapeutic antibodies targeting cell surface epitopes on MM cells. Antibody derivatives against surface glycoforms were developed and optimized for the induction of antibody-dependent cellular cytotoxicity against MM cells. Additionally, an antibody-mimetic peptide was developed, and it was used to deliver a pro-apoptotic peptide that killed MM cells. The engineered antibody derivatives show promise for the further development of cancer therapeutics.

**Abstract:**

Despite therapeutic advances in recent years, there are still unmet medical needs for patients with multiple myeloma (MM). Hence, new therapeutic strategies are needed. Using phage display for screening a large repertoire of single chain variable fragments (scFvs), we isolated several candidates that recognize a heavily sulfated MM-specific glycoform of the surface antigen syndecan-1 (CD138). One of the engineered scFv-Fc antibodies, named MM1, activated NK cells and induced antibody-dependent cellular cytotoxicity against MM cells. Analysis of the binding specificity by competitive binding assays with various glycan ligands identified N-sulfation of glucosamine units as essential for binding. Additionally, site-directed mutagenesis revealed that the amino acids arginine and histidine in the complementarily determining regions (CDRs) 2 and 3 of the heavy chain are important for binding. Based on this observation, a heavy-chain antibody, known as a nanobody, and a peptide mimicking the CDR loop sequences were designed. Both variants exhibited high affinity and specificity to MM cells as compared to blood lymphocytes. Specific killing of MM cells was achieved by conjugating the CDR2/3 mimic peptide to a pro-apoptotic peptide (KLAKLAK)_2._ In a co-culture model, the fusion peptide killed MM cells, while leaving normal peripheral blood mononuclear cells unaffected. Collectively, the development of antibodies and peptides that detect tumor-specific glycoforms of therapeutic targets holds promise for improving targeted therapies and tumor imaging.

## 1. Introduction

The current treatment strategies for cancer are still largely dominated by sole or combination of chemotherapy, radiation therapy, and surgery, all of which are associated with damage to healthy tissues or severe toxicities. Immunotherapy, a collective of treatment options that employ different means to re-engage the immune system’s anti-tumor response, has emerged as an attractive treatment option for certain types of cancer. [1,2]. This strategy is mainly driven by antibodies targeting cancer cells or immune cells. For example, the monoclonal antibodies Nivolumab and Ipilimumab, which block inhibitory signals received by T lymphocytes, thereby boosting the anti-tumor response, are now widely implemented treatment options [1]. Although the clinical benefit of immunotherapy in advanced melanoma and lung cancer is evident, the therapy is only effective in a subset of patients [3,4,5,6]. Moreover, effective immunotherapy options are lacking for many incurable blood malignancies, such as multiple myeloma (MM), acute myeloid leukemia, and myelodysplastic syndrome [7,8,9].

Multiple myeloma (MM) originates from long-lived terminally differentiated B cells or plasma cells in lymph nodes, which then colonize the bone marrow, resulting in destruction of adjacent bone tissue [10]. The disease accounts for 1% of all malignancies and is the third most common hematological malignancy. With an average age of diagnosis of 70 years, most MM patients are elderly, which generates several therapeutic challenges [11,12]. Additionally, many patients have comorbidities that often prevent hard-line therapies, such as autologous stem cell transplantation [11]. The current passive immunotherapy for MM in clinical development has focused on targeting normal cell surface receptors such as CD38, CD319, and CD138 [13,14,15,16]. Although certain antibodies targeting the core protein of these receptors were approved for MM, relapse or progression occurs in 50–60% of cases. Hence, the disease remains incurable, and patients continue to cycle through therapies, with their prognosis worsening with each relapse [12]. The major challenges are thus to identify new antibodies and their derivatives that evoke potent immune responses against MM cells. Such molecules can also be utilized as delivery agents for several potent therapeutic modalities such as antibody-drug conjugates (ADC), bispecific antibodies (BiAbs), and antibody-based chimeric antigen receptors (CARs).

In this study, we aimed to identify new human single-chain variable fragments (scFvs) against cell surface epitopes on myeloma cells. This was accomplished by affinity selection with a semi-synthetic scFv library on live MM cells, a platform that has been used for the development of therapeutic antibodies [17,18,19,20,21]. Some of the isolated scFv candidates were converted into human IgG1-Fc fusion proteins (scFv-Fc) and analyzed for specificity and efficacy to activate immune effector cells and kill MM cells. Additionally, a human heavy-chain-only antibody and a peptide mimicking the CDR loops of the variable heavy chain (VH) were designed to address certain medical needs such as drug delivery and tumor imaging.

## 2. Materials and Methods

### 2.1. Reagents

Dextran (50,000), dextran sulfate (7000–20,000), chondroitin sulfate, heparin, *N*-acetyl heparin, and trypsin-EDTA (0.25%) were purchased from Sigma-Aldrich (St. Louis, MO, USA), and the powder reagents were dissolved in sterile water. FITC- or PE-labeled anti-CD138, anti-CD4, anti-CD19, and monensin were purchased from Biolegend (San Diego, CA, USA). FITC-labeled anti-CD56 and PE-Cy5-mouse anti-human CD107a were purchased from BD Pharmingen (San Diego, CA, USA). FITC-labeled anti-human or anti-mouse IgG Fc was purchased from Sigma (St. Louis, MO, USA). PE-conjugated streptavidin was purchased from BD Biosciences (San Jose, CA, USA). Cell trace carboxyfluorescein succinimidyl ester (CFSE) was purchased from Invitrogen (Carlsbad, CA, USA). Protein A/G agarose and heparin sepharose were purchased from Santa Cruz Biotechnology (Dallas, TX, USA) and Abcam (Cambridge, UK), respectively.

### 2.2. Peptides

Synthetic peptides were chemically produced by GeneCust (Dudelange, Luxembourg) or GenScript (Rijswijk, Nederland). The identity of the peptides was verified by mass spectrometry, and the purity was determined by HPLC to be at least 85%. All peptides were dissolved in sterile water at the desired concentrations, and aliquots were stored at −80 °C until use.

MC peptide: Biotin-GRRPHGGGRRPRK-NH_2_

KLA peptide: KLAKLAKKLAKLAK-NH_2_

MC-KLA fusion peptide: GRRPHGGGRRPRKGGSKLAKLAKKLAKLAK-NH_2_

A flexible short linker (GGS) was placed between the MC peptide and the KLA lytic domain to minimize potential steric hindrance, which may block the binding to target cells.

### 2.3. Cancer Cell Lines

The cancer cell lines Ramos (CRL-1596) U266 (TIB-196), PC3 (CRL-1435), KG1a (CCL-246.1), HL60 (CCL-240), HEK293T (CRL-3216), and U937 (1593.2) were purchased from the American Type Culture Collection (Manassas, VA, USA). The multiple myeloma RPMI-8226 and CAG cell lines were obtained from Anders Aune Tveita (Institute of Immunology, Oslo University Hospital). The cells were cultured in RPMI 1640 medium or Dulbecco’s Modified Eagle’s Medium (DMEM) supplemented with 10% heat-inactivated fetal bovine serum (FBS) and 1% penicillin-streptomycin (PS).

### 2.4. Isolation of Blood Cells

PBMCs were isolated from anonymous healthy donor buffy coats provided by the Oslo University Hospital Blood Bank (project number: F8). NK cells were isolated from PBMCs using CD56-reactive microbeads and an AutoMACS Pro Separator according to the manufacturer’s instructions (Miltenyi Biotec, GmbH). Purification was verified by phenotypic analysis of the surface marker CD56. B and T cells were isolated from PBMCs using the Dynabeads™ CD19 PanB with DETACHaBEAD^®^ CD19 and CD4-/CD8-Dynabeads™ positive isolation kits, respectively, as described by the manufacturer (Invitrogen Dynal AS, Oslo, Norway). The cells were cultured in either RPMI-1640 supplemented with 10% heat-inactivated FBS and 1% PS or in X-vivo 15 medium (Lonza, Basel, Switzerland) supplemented with 1% PS. Approval for obtaining buffy coats from healthy volunteers was granted by the Oslo University Hospital Ethics Committee.

### 2.5. Affinity Selection of scFv Antibody Fragments

**Figure 1:**
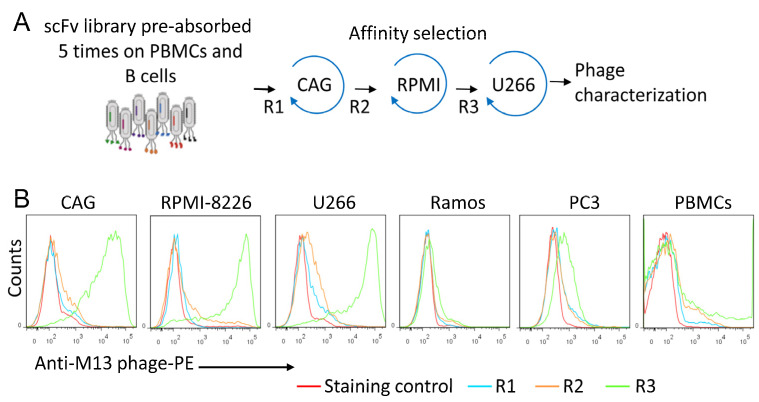
Sequential affinity selection on MM cell lines. (**A**) Schematic illustration of the screening protocol. The phage library was pre-incubated five times with peripheral blood mononuclear cells (PBMCs) from 5 different donors and normal blood B cells, and then affinity selected on the indicated multiple myeloma (MM) cell lines (CAG, RPMI 8227, U266). (**B**) Representative flow cytometry histograms showing the binding of polyclonal phage preparations from rounds 1, 2, and 3 to MM, Ramos lymphoma, and PC3 prostate cancer cell lines.

A semi-synthetic human scFv phage library was affinity selected on multiple myeloma (MM cell lines. Prior to selection, the library was pre-absorbed 5 times on PBMCs (1 × 10^8^ cells) isolated from healthy donor buffy coats. Unbound phages were further depleted on purified B cells (1 × 10^7^ cells) and the phages were sequentially affinity selected on three MM cell lines as illustrated in Figure 1A. To block the unspecific binding of the phage coat proteins with cell surface proteins, MM cells were incubated with the M13KO7 helper phage prior to affinity selection. After 1 h of incubation with the ScFv library at 4 °C, MM cells were washed 10 times in PBS supplemented with 2% BSA to eliminate unbound phages. Cell-bound phages were eluted in 300 μL 1 M Tris-glycine pH 2.2 (1% BSA), and then neutralized with 50 μL of 1 M Tris-HCl, pH 9.5. To amplify the recovered phages, 3–6 mL of exponentially growing *E. coli* TG1 cells were infected with 250 μL of the eluted phages, plated on 2xYT agar containing 100 μg/mL ampicillin and 1% glucose, followed by incubation overnight at 30 °C. Bacteria were scraped from the plates and the phages were amplified, PEG-purified, and titrated. CAG cells were used for the first round of affinity selection. For the second round, the amplified CAG-bound phages were affinity selected on RPMI-8226 cells as described above. RPMI-8226-bound phages were eluted, amplified, and then affinity selected on U266 cells. Subsequently, U266-bound phages were eluted, propagated, and then tested for binding to the three used MM cell lines. After the third selection round, single phage clones were randomly picked, and scFv phage antibodies were produced and tested for binding to MM cells and blood B cells using flow cytometry. Phages that bound to the three cell lines but not blood B cells were selected, and their DNA sequences were determined using the pHEN primer 5′-CTATGCGGCCCCATTCA-3′ (NWG, Germany). The phage scFv library used in this study was a generous gift from Dr. Greg Winter (University of Cambridge, Cambridge, United Kingdom).

### 2.6. Phage Amplification

Phage amplification and precipitation were performed as previously described [20,21]. In brief, exponentially growing *E. coli* strain TG1 cells were infected with the eluted phages and grown in 2xYT medium containing 100 μg/mL ampicillin and incubated for about 1 h at 37 °C with shaking. The cells (12 mL) were then infected with the M13KO7 helper phage (Life Technologies, Bleiswijk, The Netherlands). After infection, cells were cultured overnight at 30 °C in 100 mL of 2xYT medium containing 100 μg/mL ampicillin and 50 μg/mL kanamycin for the selection of M13KO7-infected bacteria. The overnight culture was centrifuged at 9000× *g* for 10 min, and the phage particles were precipitated by adding 1/5 volume of 20% polyethylene glycol (PEG) 6000/2.5 M NaCl and incubated for a minimum of 2 h or overnight at 4 °C. After incubation, the samples were centrifugated at 11,000× *g* for 30 min, and the phage pellets were dissolved in PBS and stored at 4 °C. Phages were also prepared from individual ampicillin-resistant colonies, PEG-precipitated, titrated, and their binding to MM cells was evaluated by flow cytometry.

### 2.7. Cloning and Expression of the Selected scFv–Fc Fusion Proteins

Synthetic genes coding for selected scFvs (VH-linker-VL) with *EcoR1* and *BglII* restriction sites at the 5′ and 3′ ends, respectively, were made by GenScript (Rijswijk, Nederland). *EcoR1*/*BglII*-digested product was cloned into an *EcoR1*/*BglII*-digested pFuse-hIgG1-Fc2 vector in frame with the IL-2 signal sequence and the Fc portion of human IgG1 (In vivoGen, San Diego, CA, USA). The MM1 gene was also cloned in the *EcoR1*/*BglII*-digested pFuse-hIgG1-e5-Fc2 vector carrying the triple mutation (S239D/I332E/A330L, DEL), known to enhance antibody-dependent cellular cytotoxicity (ADCC) and antibody-dependent cellular phagocytosis (ADCP) by increasing the affinity for Fcγ RIIIa. Positive clones were selected and verified by DNA sequencing. Recombinant scFv-Fc fusion proteins were produced by transient transfection of the plasmids into HEK293T cells using LipofectMax (ABP Biosciences, Beltsville, MD, USA). After 72 h, supernatants from transfected cells were collected, and the scFv-Fc fusion proteins were affinity purified with gravity-flow protein A/G column chromatography. The eluted protein fractions were analyzed by sodium dodecyl sulfate–polyacrylamide gel electrophoresis (SDS-PAGE) using 10% polyacrylamide gels, followed by Coomassie staining with Imperial™ protein stain (Thermo Fisher Scientific, Rockford, IL, USA). Positive fractions were collected, pH adjusted to 7.5, and the ScFv-Fc concentrations determined with NanoDrop. Samples were stored at −20 °C until use. The same protocols were used for the cloning and expression of the heavy-chain-only antibody.

### 2.8. Flow Cytometry Analysis

The binding of polyclonal and monoclonal scFv phage clones to cells was determined by flow cytometry. In brief, aliquots of cells (10^5^) were plated in a conical 96-well microplate, washed in PBS buffer containing 2% FBS, and then incubated with ScFv-phages for 30 min on ice. After washing, the cells were incubated with biotin-conjugated anti-M13 monoclonal antibody followed by PE-conjugated streptavidin. Stained cells were analyzed by flow cytometry (Canto II, BD Biosciences). Similarly, the binding of the scFv-Fc fusion proteins to different cell types was detected by flow cytometry analysis using fluorescein isothiocyanate (FITC)-conjugated anti-IgG Fc-specific antibodies of mouse or human species reactivity. In brief, 2–5 × 10^5^ cells/100 µL/sample were incubated with the desired mouse or human antibodies for 40 min at 4 °C, followed by staining with goat anti-mouse IgG Fc cross-adsorbed secondary antibody FITC (Invitrogen™, Carlsbad, CA, USA) or anti-human IgG (Fc specific) FITC (Sigma-Aldrich, Saint-Louis, MO, USA) for 30 min at 4 °C. The cells were washed in FACS buffer (PBS + 0.5% BSA + 3 mM EDTA) between each incubation step. For trypsin inhibition, the cells were treated with Trypsin-EDTA (0.25%) (Life Technologies) for 5 min and washed two times in complete medium prior to staining with the MM1 ScFv-Fc and analysis by flow cytometry. Similarly, the cells were stained with biotinylated MC peptides, followed with streptavidin-PE, and processed as above. Analysis was performed on a BD FACS Canto II using the BD FACSDiva™ 6.1.3 software (BD Biosciences, San Jose, CA, USA). The obtained data was analyzed with FlowJo version 7.6.1 (FlowJo LLC, Ashland, OR, USA).

### 2.9. ELISA

Affinity measurements by ELISA for cell surface antigens were performed as previously described [22]. Briefly, U266 cells (2 × 10^5^/200 μL staining buffer) were mixed with various concentrations of the MM1 scFv-Fc (1 to 500 ng/mL) and incubated for 1 h at 4 °C with rotation before cells were pelleted by centrifugation. Supernatants were removed and retained for the quantification of unbound MM1 scFv-Fc using the human IgG ELISA Quantification Kit (Bethyl Laboratories, Montgomery, TX, USA). The concentrations of bound MM1 scFv-Fc were obtained by subtracting unbound molecules from the initial scFv-Fc concentrations, and the obtained values were used to create Scatchard plots for determining the affinity.

### 2.10. Competition Experiments

For ligand inhibition experiments, the MM1 scFv-Fc was pre-incubated with different concentrations of dextran, dextran sulfate, chondroitin sulfate, *N*-sulfated heparin, or *N*-acetyl heparin for 20–30 min prior to adding the cells. In some experiments, the cells were first incubated with the competitors and washed twice before adding the scFv-Fc fusion proteins. The direct binding of the MM1 scFv-Fc to heparin sepharose was performed as follows. Briefly, 10 μg of the MM1 scFv-Fc was incubated with heparin sepharose beads in PBS buffer for 20 min at RT with agitation. Subsequently, the beads were washed 5 times with PBS buffer to remove unbound molecules, and bound scFv-Fc molecules were acid eluted, neutralized, and then analyzed by SDS-PAGE, followed by Coomassie staining with Imperial™ protein stain (Thermo Fisher Scientific, Waltham, MA, USA). As a positive control, the same amount of the scFv-Fc was incubated with protein A/G agarose and processed as above.

### 2.11. Confocal Microscopy Analysis

CAG cells were stained with the MM1 scFv-Fc in conjunction with anti-CD138-PE under flow cytometry staining conditions. Subsequently, the cells were incubated with Hoechst 33342 (Invitrogen Dynal AS, Oslo, Norway) for 5 min, washed, and then one drop of each cell suspension was spotted onto microscope glass slips and fixed with 4% paraformaldehyde for 20 min at RT. After washing, the slides were covered with Dako cytomation fluorescent mounting medium and processed for confocal microscopy imaging. For peptide internalization, the cells were incubated with biotinylated MC peptide for 40 min at 4 °C. After washing, the cells were incubated with phycoethrin (PE)-conjugated streptavidin for 30 min at 4 °C, washed, and then incubated or not at 37 °C for 60 min to allow internalization of the bound peptide–streptavidin–PE complexes. In some experiments, the cells were incubated at 37 °C for various time intervals. To visualize the nuclei, Hoechst 33,342 was added to the cells for 5 min. After washing with PBS, one drop of each cell suspension was spotted onto microscope glass slips and processed as above. Images were acquired with a Zeiss LSM 710 confocal microscope (Carl Zeiss Microscopy GmbH, Jena, Germany), equipped with a Plan-Apochromat 63x/1.40 oil objective using pinhole size 1 Airy unit (1 AU). Images were processed with the ZEN lite software (Carl Zeiss Microscopy GmbH, version 3.3) and ImageJ (U. S. National Institutes of Health, Bethesda, MD, USA, version 1.53e).

### 2.12. Analysis of NK Cell Degranulation and Activation

NK cells were analyzed by flow cytometry for the detection of CD107a as a surrogate marker for degranulation and NK activation. Briefly, NK cells (5 × 10^4^/200 μL X-vivo 15 medium) were incubated with cancer cells coated with MM1 scFv-Fc or Fc control for 5 h at 37 °C. During stimulation, PE-Cy5-conjugated anti-CD107a (2 μL/well) and monensin (0.2 μL/well) were added to the cell cultures. After incubation, the cells were harvested, washed, stained with FITC-conjugated anti-CD56, and then run on a BD FACS Canto II flow cytometer (BD Biosciences, San Jose, CA, USA). The obtained data were analyzed with FlowJo software.

### 2.13. Cytotoxicity Assay

Target cell lysis was identified by lactate dehydrogenase (LDH) release assays according to the manufacturer’s instructions (Promega, Madison, WI, USA). Briefly, target cells and control cells were incubated with the MM1 ScFv-Fc (1 μg/mL) for 1 h at RT in X-vivo 15 medium. After incubation, the cells were washed to remove unbound antibody and plated at 15,000 cells per well in 96-well plates. Effector cells were added at an effector:target (E:T) ratio of 20:1. Plates were incubated at 37 °C for 18 h before the supernatants were collected and LDH contents determined. As a positive control (maximum release), some wells were treated with the 5% triton-X provided in the kit. The percentage of specific cell lysis was calculated as follows:

$$\frac{Experimental\;release-Background\;release}{Maximum\;release-Background\;release}\times 100$$

The cytotoxicity of the MC-KLA fusion peptide was evaluated by propidium iodide staining.

### 2.14. Syndecan-1 Knockdown and Overexpression in HEK293T Cells

Syndecan-1 expression was downregulated by RNA interference. Briefly, cells were transfected with siRNAs by electroporation using the amaxa electroporation system as previously described [23]. The used siRNAs have the following sequences: siRNA1 (5′-CACCUGGCAUCGCACCAU-3′), siRNA2 (5′-GCAGGUGCUUUGCAAGAUAU-3′), and control siRNAs (5′-GGGUUAGCGUAAUCUAACC-3′). The plasmid encoding human syndecan-1 tagged ORF clone (RC200419) was purchased from ORIGENE (Rockville, MD, USA). HEK293T cells were transiently transfected with the plasmid using lipofectamine 2000 for 72 h. After transfection, the cells were stained with PE conjugated to monoclonal 9E10 that detects the myc tag sequence (Thermo Fisher, Oslo, Norway) or with MM1 scFv-Fc.

### 2.15. Statistical Analysis

Results are reported as the means ± SD. The statistical significance of differences was assessed by Student’s *t*-test. Anti-tumor effects of MM1 versus Fc control were assessed by a two-tailed test. The level of significance was set at a *p* value of less than 0.05. All experiments were performed at least three times if not otherwise indicated. Differences between control and treated cells were measured by a standard Student’s *t*-test. For multiple comparisons, a two-way ANOVA analysis was used. *p* values < 0.05 were considered significant.

## 3. Results

### 3.1. Isolation of MM-Specific scFv Antibody Fragments

Despite advances in recent years, there are still unmet medical needs for patients with multiple myeloma (MM). A key challenge in the improvement of patient outcomes is the development of therapeutics that allow effective and specific targeting of malignant plasma cells. We have established a whole-cell screening strategy to isolate new single-chain variable fragments (scFvs) that specifically/preferentially bind MM cell lines. The human scFv phage library was first pre-incubated five times with peripheral blood mononuclear cells (PBMCs) from five different donors, followed by blood B cells to remove phages that bind to common receptors. Second, the pre-absorbed library was sequentially affinity selected on three different MM cell lines (Figure 1A). Prior to affinity selection, the MM cells were pre-incubated with the helper phage to block the binding of the phage coat proteins to the cells. Different cancer cell lines were used in the selection process to increase the chance of selecting phage scFvs that bind to common receptors or carbohydrate/lipid structures preferentially or exclusively expressed by MM cells. After the third round of affinity selection, polyclonal phages were tested for binding to the used MM cell lines, normal peripheral blood mononuclear cells (PBMC), Ramos lymphoma cell line, and PC-3 prostate cancer cell line. A significant enrichment (around an 800-fold increase) of phage binding to MM cells was obtained after the 3rd round of affinity selection (Figure 1B, Table 1). Enrichment factors were calculated as output versus input ratios. Thus, our selection protocol resulted in the enrichment of phage clones with specific binding to MM cells. The binding to other cell types tested was insignificant.

**Figure 2:**
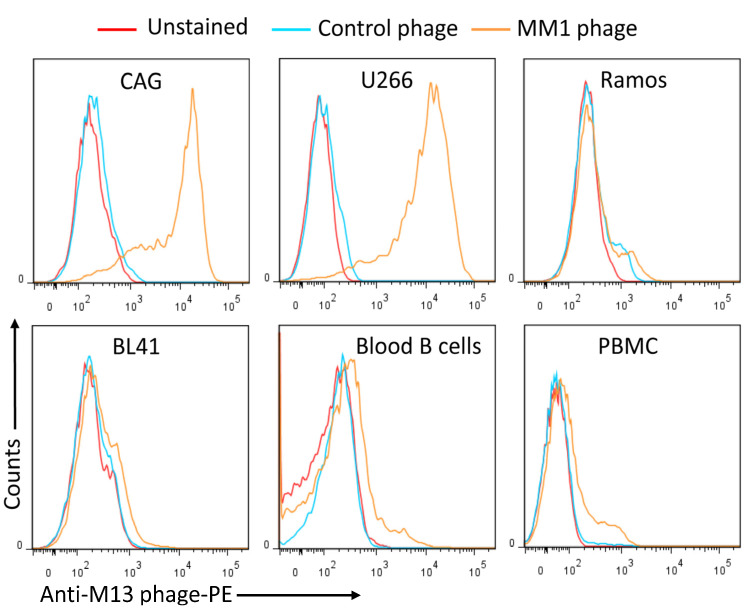
Binding of monoclonal phage clones. Representative flow cytometry histograms showing the binding of the MM1 scFv phage clone to multiple myeloma (CAG, U266), Ramos, and BL41 lymphoma cell lines. Normal blood B cells and peripheral blood mononuclear cells (PBMC) are also included. Cells were stained with the indicated phages, followed with an anti-M13 monoclonal antibody, and then analyzed by flow cytometry. The presented data are from one single experiment and representative of 6 independent experiments.

Next, monoclonal scFvs were prepared from individual phage clones, and their binding to MM cell lines was investigated. Around 80% of the phage clones bound to MM cells. The binding profiles of single phage clones to MM cell lines, blood B cells, and lymphoma cell lines were evaluated by flow cytometry, and representative examples are shown in Figure 2. Most of the scFv phage clones bound strongly to MM cells, whereas no significant binding to B cells or lymphoma cell lines was observed. Sequence analysis of 30 positive phage clones identified three major sequences, with an overrepresentation of the sequences displayed by phages MM1 and MM12 (Table 2).

### 3.2. Conversion of the MM1 scFv into a scFv-Fc Antibody

**Figure 3:**
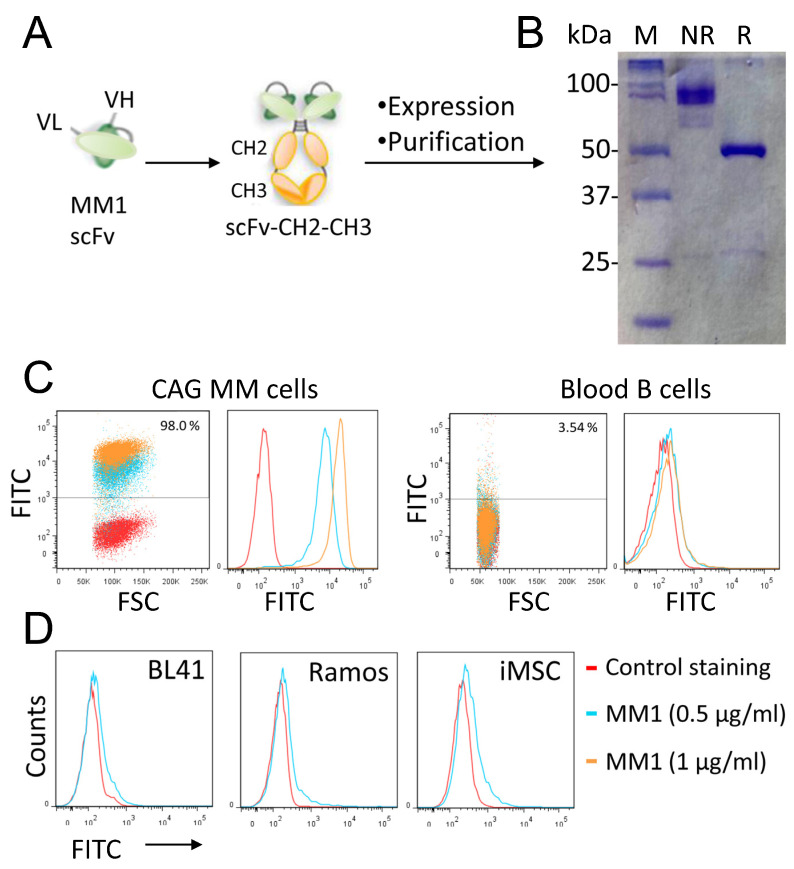
Expression and characterization of the MM1 scFv-Fc antibody. (**A**) Schematic diagram showing the conversion of the MM1 scFv into an scFv-Fc fusion protein. (**B**) An SDS-PAGE analysis of purified MM1 scFv-Fc under non-reducing (NR) and reducing (R) conditions. (**C**,**D**) Binding of the scFv-Fc to cancer cell lines and normal blood B cells. The cells were stained with the recombinant MM1 scFv-Fc and then analyzed by flow cytometry. The data are from one single experiment and are representative of five independent experiments.

Based on its favorable binding profile, the phage scFv named MM1 was selected for further studies. The MM1 sequence was fused to the human IgG1 Fc domain to generate an scFv-Fc fusion antibody (CH3-CH2-hinge-scFv fusion) for functional analysis (Figure 3A). After cloning and verification, the MM1 scFv-Fc was expressed in HEK293T cells, purified with protein A/G agarose affinity columns, and then analyzed by SDS-PAGE (Figure 3B). Under reducing conditions (R), the scFv-Fc migrated as a 50 kDa monomer, whereas under non-reducing conditions, it migrated as a band of 100 kDa, corresponding to the dimeric structure of the scFv-Fc antibody. Similar to the phage clone, the antibody bound with high affinity (around 3 nM) to MM cell lines. Figure 3C shows the binding of the scFv-Fc to the CAG MM cell line and freshly isolated normal blood B cells. At the concentration used, the MM1 scFv-Fc displayed no significant binding to normal B cells. Similarly, scFv-Fc did not bind to lymphoma cell lines (Ramos, BL41) and immortalized mesenchymal stem cells (Figure 3D). 

### 3.3. Activation of NK Cells and Induction of ADCC against Myeloma Cell Lines

**Figure 4:**
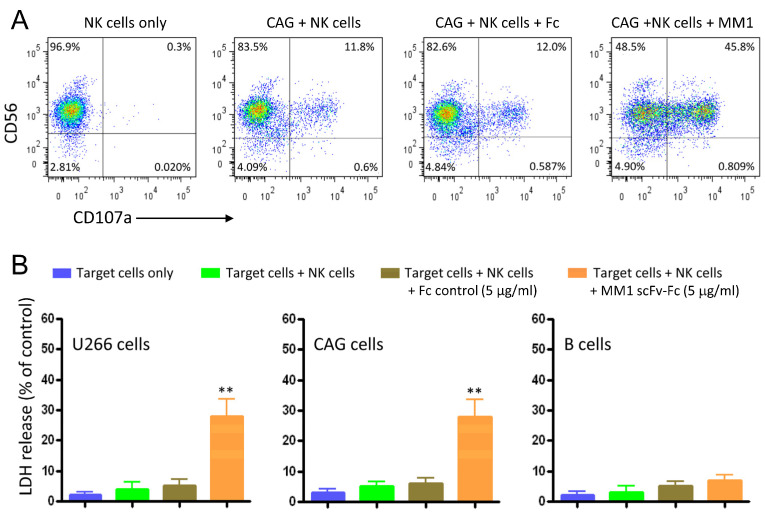
Induction of NK cell degranulation and ADCC by the MM1 ScFv-Fc. (**A**) Surface display of the CD107a degranulation marker on NK cells. The CAG MM cell line was incubated with the MM1 scFv-Fc or Fc control for 30 min at room temperature. Thereafter, the cells were washed to remove unbound molecules and cultured in X-VIVO 15 medium for 5 h at 37 °C. CD107a surface display was analyzed by flow cytometry using a specific monoclonal antibody. Results are from one single experiment and are representative of three independent experiments using NK cells from different donors. The numbers in dot plots represent the percentage of CD107a-positive NK cells. (**B**) ADCC as measured by LDH release. The indicated target (T) cells (U266, CAG, B cells) were pre-incubated with the MM1 scFv-Fc or Fc control for 30 min in X-VIVO 15 medium. After washing, freshly isolated effector (E) NK cells were added to the cells at E/T ratio of 20:1. After 18 h incubation at 37 °C, culture supernatants were collected and analyzed for LDH. Statistically significant differences between the MM1- and the Fc control-treated cells are indicated by asterisks. Results are represented as means ± SD from triplicate determinations and are representative of three independent experiments using NK cells from different donors. ** *p* < 0.01.

Cytotoxic lymphocytes are important for immune responses against viral infections and cancer. We therefore investigated whether MM1 scFv-Fc-coated MM cells could induce the activation of NK cells. As illustrated in Figure 4A, the MM1 scFv-Fc promoted a significant degranulation in the presence of CAG MM cells when compared to the Fc control (45% vs. 12%). In the context of antibody treatment, NK cells are unique in that they express only the low-affinity activating FcγR CD16 (FcγRIIIA) and no inhibitory receptors, which underscores the significance of antibody-dependent cellular cytotoxicity (*ADCC*) in eliminating tumor cells [24]. Next, we assessed the cytolytic activity of the recombinant MM1 scFv-Fc using the LDH release assay. CAG and U266 cell lines were used as target cells and human NK cells as effector cells. Figure 4B presents a significant increase (*p* < 0.01) of cytolysis by the MM1 scFv-Fc at an effector-to-target cell ratio of 20:1, revealing 29% (±5%) cytotoxicity of CAG and 23% (±5%) of U266. The ADCC effect was antigen-specific, as normal B cells were not affected.

### 3.4. Evidence for the Interaction of the scFv-Fc with Cell-Surface Heparan Sulfate

**Figure 5:**
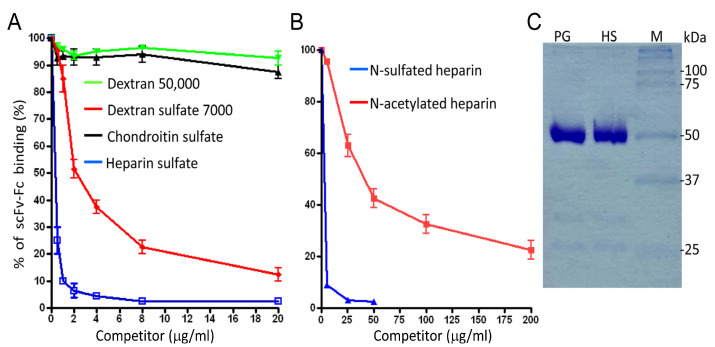
Inhibition of MM1 scFv binding by glycan ligands. (**A**,**B**) competition assays. The MM1 scFv-Fc (1 μg/mL) was incubated with the indicated competitors for 20 min at RT prior to the addition U266 MM cells and FACS analysis as described under Materials and Methods. Percent inhibition of binding is shown as a function of increasing competitor concentration. The inhibition was calculated from geometric mean fluorescence intensity. Average mean values (±SD) of three independent experiments are shown. (**C**) Affinity capture of the scFv-Fc using protein A/G agarose (PG) or heparin sepharose beads (HS). The same amount of antibody (10 μg) was incubated with the indicated beads for 20 min at RT, washed to remove unbound molecules before scFv-Fc molecules were acid eluted in 20 μL buffer. The eluates (10 μL/sample) were separated by SDS-PAGE prior to Coomassie staining.

The presence of positively charged amino acids within the heavy chain CDR2 and CDR3 regions (Table 2) prompted us to investigate whether the MM1 scFv-Fc antibody recognizes negatively charged cell surface ligands. Therefore, we tested whether polyanionic ligands could inhibit the binding of MM1 scFv-Fc to MM cells. Pre-incubation of the scFv-Fc with dextran sulfate or heparin, both polysulfated polysaccharides, inhibited the binding (Figure 5A). These results would first indicate that the negative charge of the polysaccharide plays a significant role in blocking the scFv-Fc binding, as uncharged dextran failed to inhibit binding even at high concentrations (Figure 5A). Second, sulfation as such is not sufficient to create the MM1 epitope because chondroitin sulfate (CS) is not immunoreactive (no inhibition). The glucosamine residues (GlcN) of CS are sulfonated at the C-6 and/or C-4 positions, whereas heparin displays *O*-sulfonation at the C-2 of the uronic acid and the C-6 of GlcN [25,26]. Hence, the scFv-Fc seems to bind to 2-*O*-sulfonated disaccharide units rather than their 6-*O*-sulfonated counterparts. The used heparin also contains sulfo groups at the N-position of GlcN. Interestingly, heparin with N-sulfo groups is a much more potent competitor for MM1 than its counterpart with N-acetyl groups (Figure 5B). The half maximal inhibitory concentration (IC50) for N-acetylated heparin is around 40 μg/mL, whereas that of N-sulfated heparin is less than 0.20 μg/mL. Therefore, the presence of an N-sulfate resulted in a 200-fold increase in affinity, almost representing an on/off switch for binding.

When cells were pre-incubated with heparin and washed before adding the MM1 scFv-Fc, no inhibition was observed [27], indicating that the inhibition was due to the interaction between the scFv-Fc antibody and heparin. Using heparin sepharose beads as a binding matrix, we confirmed the direct interaction of the antibody and heparin (Figure 5C). Moreover, the inhibition of protein sulfation using sodium chlorate [28] inhibited the binding of the scFv-Fc to the MM cell line RPMI-8226 [27].

### 3.5. Involvement of Syndecan-1 Associated Heparan Sulfate

**Figure 6:**
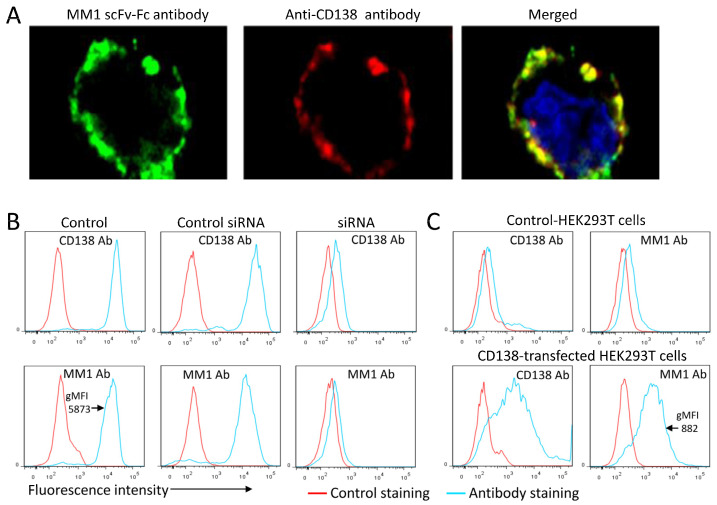
Identification of HS syndecan-1 as a potential receptor. (**A**) Confocal microscopy images showing the co-localization of the MM1 scFv-Fc and syndecan-1 core protein. CAG cells were co-stained with the indicated antibodies and then analyzed by confocal microscopy to check for surface binding. Nuclei were visualized with Hoechst 33342 staining (blue). The data are representative of three independent experiments. (**B**) Silencing of syndecan-1 expression in U266 inhibited the binding of MM1 scFv-Fc antibody (MM1 Ab), whereas overexpression of syndecan-1 in HEK293T enhanced its binding (**C**).

Among the members of the integral membrane family of heparin sulfate proteoglycans, syndecan-1 (CD138) is highly expressed on MM cells and could be participating in MM1 binding [29]. The clinical relevance of this target is universally valued through its routine use as a diagnostic marker in multiple myeloma [30]. In the first experiment, we used microscopic imaging to determine the distribution of the MM1 receptor and syndecan-1 on MM cells. We co-stained the cells with MM1 scFv-Fc (green) and an anti-CD138 monoclonal antibody (red) at 4 °C and subsequently imaged the cells by confocal microscopy (Figure 6A). A high proportion of the MM1 antibody–receptor complexes did co-localize with syndecan-1 (yellow).

To further confirm the involvement of syndecan-1 in antibody binding, we knocked down its expression in U266 cells by transfection with syndecan-1-specific siRNAs. The expression of syndecan-1 was significantly downregulated in siRNA-transfected U266 cells as compared to controls (Figure 6B upper panels). MM1 binding to U266 cells with syndecan-1 knockdown was also inhibited compared to controls (Figure 6B, lower panels). To further support these results, we also examined whether syndecan-1 overexpression in HEK293T cells would confer scFv-Fc binding. Enhanced MM1 scFv-Fc antibody binding was observed in syndecan-1 overexpressing HEK293T cells (Figure 6C). The binding of the MM1 antibody to HEK293T cells overexpressing syndecan-1 was around 6.6-fold weaker than that seen with MM cells, suggesting that the heparan sulfate is highly modified in MM cells. In summary, silencing of syndecan-1 expression in U266 cells inhibited MM1 scFv-Fc antibody binding, whereas its overexpression in HEK293T cells significantly increased the binding. Of note, the binding of MM10 and MM12 antibodies to MM cells was inhibited by heparin sulfate but not by chondroitin sulfate. Since syndecan-1 is the dominant, if not the only, heparan sulfate proteoglycan expressed on MM cells, the data would indicate that these antibodies also target the heparan sulfate on the syndecan-1 protein (Supplementary Figure S1). Collectively, these results support the importance of heparan sulfate on the syndecan-1 core protein in antibody binding.

### 3.6. The Role of the CDRs of the scFv-Fc Heavy Chain in Recognition of MM Cells

**Figure 7:**
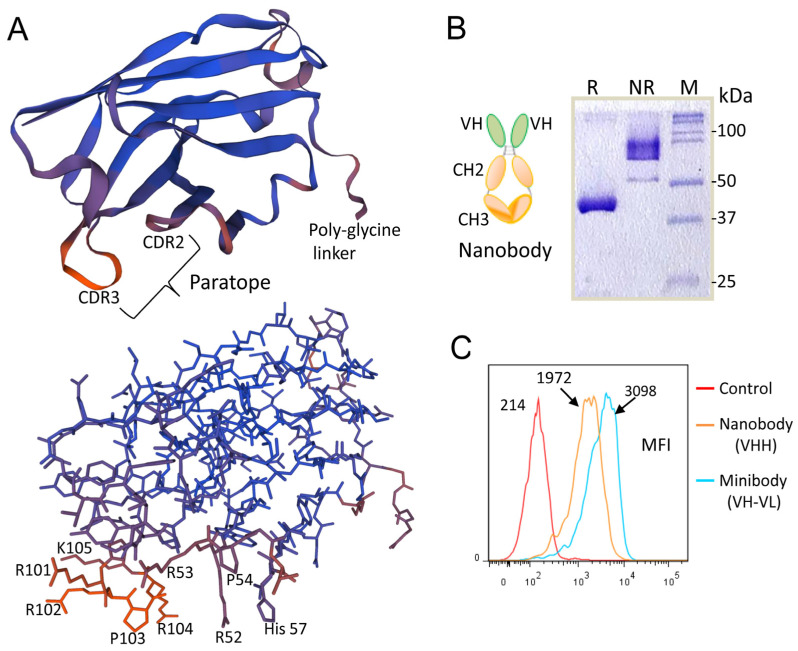
Characterization of the heavy-chain-only nanobody. (**A**) Predicted 3D structure of the heavy-chain-only nanobody. Structure modeling was performed using the accessible Expasy web server (https://swissmodel.expasy.org, accessed on 10 December 2021). The CDR2 and CD3 loop sequences and positive charge amino acids are indicated. R, arginine; K, lysine; His, histidine; P, proline. (**B**) Conversion of the VH into nanobody (VH-hinge-CH2-CH3) and analysis of the purified nanobody by SDS-PAGE under reducing (R) and non-reducing (NR) conditions. (**C**) Representative histograms showing the binding of the nanobody to MM cells. The cells were stained with the recombinant nanobody (10 nM) and then analyzed by flow cytometry. For comparison, the cells were also stained with MM1 scFv-Fc (10 nM). The data derive from one single experiment and are representative of three independent experiments.

As the amino acid sequence of the complementarity-determining regions (CDRs) of an antibody is a key determinant of antigen specificity [31], we performed site-directed mutagenesis on these regions to identify the residues responsible for antigen binding. Alanine replacement was introduced into the CDRs of MM1 and MM12 scFv-Fc antibodies prior to analysis of the binding affinity of the mutant variants. We found that the amino acids arginine and histidine, present in the CDR2 and CDR3 of the heavy chain, are important for the scFv-Fc binding to MM cells. Given that the heavy chain CDR2 and CDR3 are the major determinants of the scFv-Fc binding, we sought to investigate whether an scFv-Fc with a heavy chain only, known as a nanobody, can be designed. The VH domain of this nanobody contains a consensus sequence of the heavy chain CDR2 (RRPGLH) and CDR3 (AKGGRRPRK) in MM1, MM10, and M12 scFvs (see Table 2). Since nanobodies have extended CDR3 lengths compared to those of conventional antibodies, we thought that the use of a longer amino acid sequence (e.g., consensus sequence) may facilitate the binding of the nanobody to MM cells. The predicted 3D structure of the single-variable domain VH is shown in Figure 7A. All the amino acid residues in the CDR3 and CDR2 loops are surface exposed and therefore ready for interacting with surface heparan sulfate (lower panel).

A synthetic gene encoding the VH chain was genetically fused to the human IgG1 Fc domain (hinge, CH2-CH3) to generate a nanobody that was produced in HEK293T cells. SDS-PAGE analysis revealed that the nanobody is of the expected size for a monomer (40–45 kDa) under reducing conditions and of a dimer (80–90 kDa) under non-reducing conditions (Figure 7B). The engineered nanobody retained a high affinity for MM cell lines (Figure 7C, as a representative example). The heavy chain of both MM1 and MM12 scFv is derived from the germline DP-47/VH3-23, which is the most frequently used VH gene segment in peripheral blood B cells [32]. Although more work is needed, these results suggest that the VH3-23 gene segment can be used to generate human nanobodies. It should be noted that the camelid heavy-chain-only antibodies display a high degree of sequence similarity with human VH3 domains [33].

### 3.7. Rational Design of a Peptide That Mimics the Selected Heparan Sulfate scFvs

**Figure 8:**
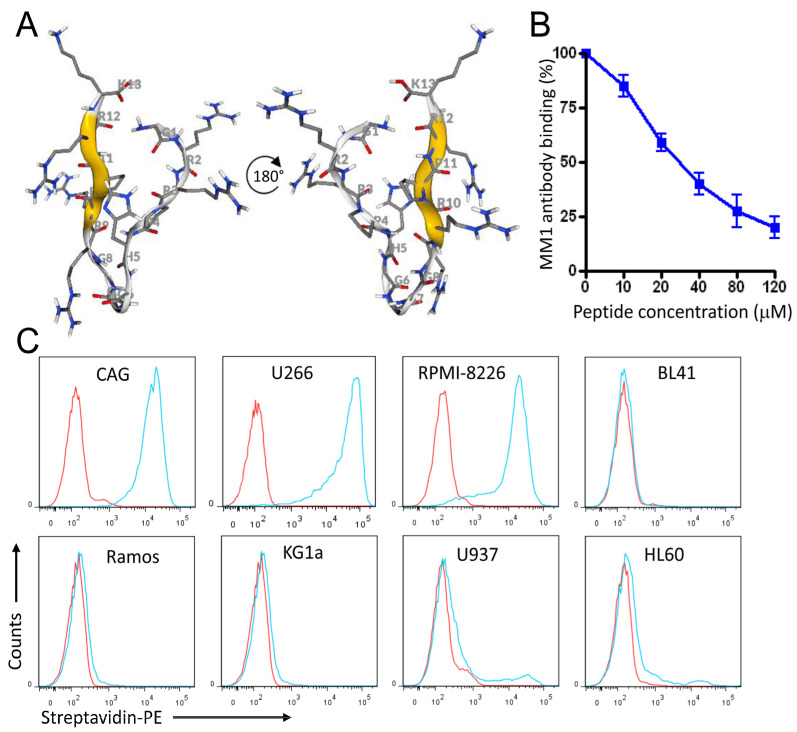
Characterization of the MC mimicking peptide. (**A**) The predicted 3D structure of the MC peptide. Structure modeling was performed using the program Pep-Fold (http://bioserv.rpbs. univ-parisdiderot.fr/PEP-FOLD, accessed on 12 April 2022). (**B**) Inhibition of MM1 scFv-Fc binding by the MC peptide. U266 cells were incubated with various concentrations of the peptide prior to the addition of the MM1 scFv-Fc (10 nM) and analysis by flow cytometry. Percent inhibition of binding is shown as a function of increasing peptide concentration. The mean of three experiments is shown. (**C**) Representative flow cytometry histograms showing the binding of the MC peptide (blue) to MM (CAG, U266, RPMI-8226), lymphoma (Ramos, BL41), and leukemia (KG1a, U937, HL60) cell lines.

**Figure 9:**
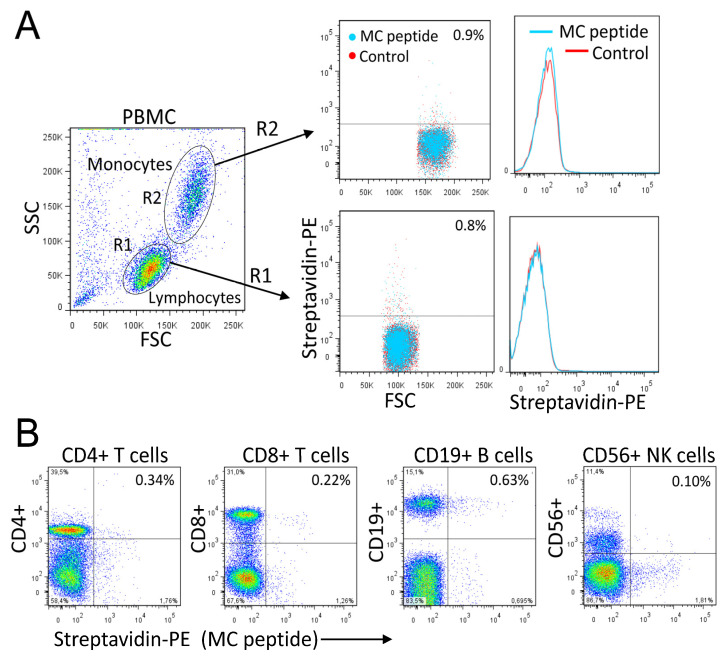
Binding of the MC peptide to blood mononuclear cells. (**A**) PBMCs were incubated with biotinylated MC peptide (2 μg/mL), followed by streptavidin-PE and analysis by flow cytometry. Both histograms and dot blots are shown. The gated cell populations are indicated. (**B**) PBMC were co-stained with the biotinylated MC peptide (2 µg/mL) and fluorescently labeled monoclonal antibodies against CD4, CD8, CD19, or CD56. Then, the cells were analyzed by flow cytometry. The percentage of double-stained cells was insignificant.

**Figure 10:**
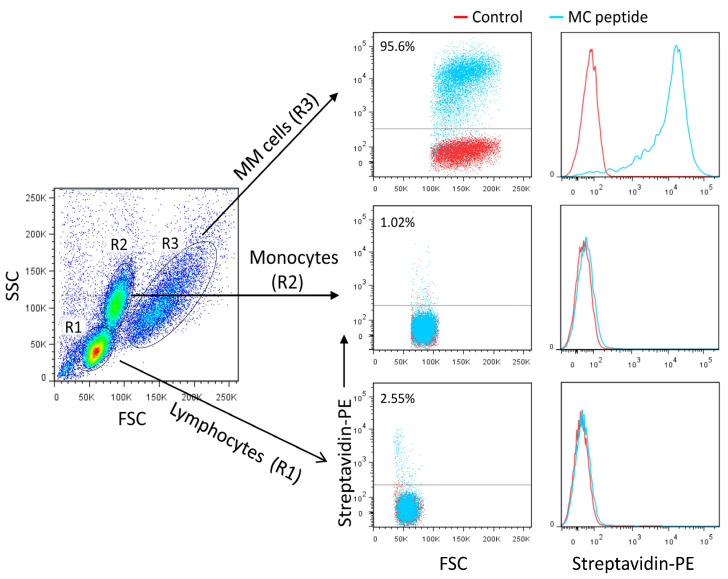
Analysis of the MC peptide specificity. PBMC were mixed with U266 MM cells, stained with the MC peptide (2 μg/mL), and analyzed by flow cytometry. Histograms and dot plots are shown. Gated cell populations are indicated. Only MM cells were stained with the MC peptide.

The success in generating nanobodies encouraged us to explore the feasibility of engineering even smaller ligands against heparan sulfate. Smaller peptide ligands represent promising reagents by retaining high affinity and overcoming challenges such as tumor penetration. By performing structural analysis of the scFv-Fc fusion proteins and nanobody CDR2-CDR3 loops, a candidate peptide, named MC, likely to mimic the scFv-Fc-heparan sulfate interactions was designed (GRRPHGGGRRPRK). The predicted 3D structure of the MC peptide is shown in Figure 8A. The peptide competed with the MM1-scFv-Fc binding to MM cells (Figure 8B). Additionally, it recognized MM cells with high specificity and affinity when compared to other tested cancer cells (Figure 8C, as representative examples). The MC peptide did not bind to normal blood mononuclear cells, including monocytes, T cells, B cells, and NK cells (Figure 9A,B). To further demonstrate the specificity of the MC peptide, we mixed U266 MM cells with blood cells and showed that the peptide binds malignant MM cells, but not normal blood cells (Figure 10).

### 3.8. A Pro-Apoptotic Peptide Conjugated to MC Peptide Selectively Kills MM Cells

**Figure 11:**
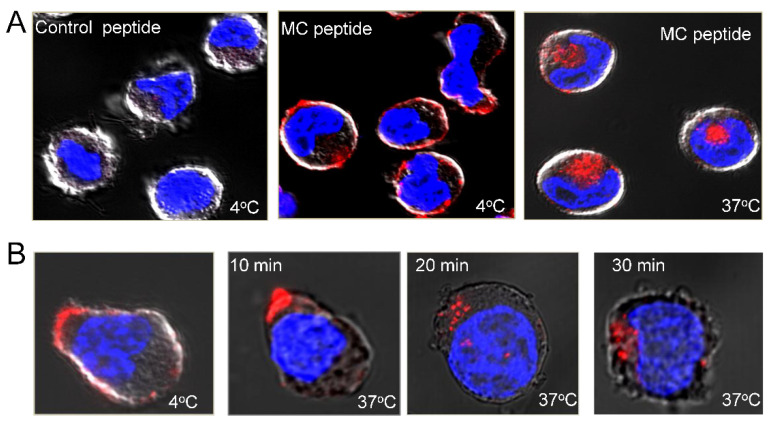
Internalization of the MC peptide–streptavidin–PE complexes by MM cells. (**A**)The CAG MM cell line was stained with biotinylated MC peptide or control peptide (2 μg/mL each), washed, and incubated with streptavidin-PE for 30 min at 4 °C. After washing, the cells were incubated at 4 °C or 37 °C for 60 min and then processed for confocal microscopy analysis. (**B**) MC peptide-stained cells were also incubated at 37 °C for 10 min, 20 min, and 30 min prior to processing. Nuclei were visualized with Hoechst 33342 staining (blue). Phase contrast and fluorescence images were obtained with a Zeiss LSM 710 confocal microscope using the Plan-Apochromat 63x/1.40 oil objective. Phase contrast and fluorescence images were merged.

Given its preferential binding to MM cells, the MC peptide could be a promising delivery agent for targeting malignant cells. Towards this goal, we first tested the ability of the peptide to promote the internalization of PE-conjugated streptavidin. The cells were incubated with biotinylated MC peptide at 4 °C, washed, and then incubated with PE-conjugated streptavidin. After washing, they were incubated either at 4 °C or 37 °C to allow internalization and then processed for confocal imaging (Figure 11A). No fluorescent signal is obtained for cells stained with the control peptide/strep-PE and incubated at 4 °C. By contrast, cells stained with the MC peptide in combination with streptavidin-PE displayed surface staining. Additionally, the cells internalized streptavidin-PE-bound MC peptide when incubated at 37 °C, thus opening the possibility of delivering even large payloads to MM cells (Figure 11A). Interestingly, most of the peptide–PE complexes are rapidly internalized into MM cells (Figure 11B). Of note, the MC peptide displays no cytotoxicity on cultured cell lines or normal peripheral blood cells.

**Figure 12:**
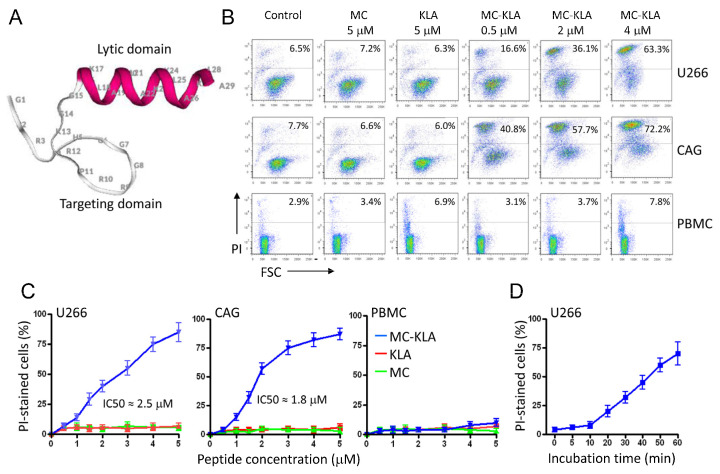
Characterization of the lytic fusion peptide. (**A**) The 3D structure of the MC-KLA fusion peptide. (**B**) Quantitative cytotoxicity assay using flow cytometry. Each cell line was treated with the indicated peptide concentrations for 60 min at 37 °C in RPMI medium supplemented with 5% FCS prior to cell viability assessment by PI staining and flow cytometry. Freshly isolated peripheral blood mononuclear cells (PBMCs) were included as normal cells. Representative dot plots are shown. A summary of the cell viability data is shown in (**C**). The mean ± SD of three experiments is shown. The IC50 values were calculated as the concentration of the peptide required for 50% reduction in cell viability. (**D**) Time course cell viability. U266 cells were incubated with peptides (3 μM) and the cell viability was quantitated at various time points using PI staining. The data represent the mean ± SD of three independent experiments.

To develop a targeted agent for killing MM cells, we next chemically fused the MC peptide to the KLA peptide as a cytotoxic drug. The peptide KLA (KLAKLAK)_2_-NH_2_, which is rather non-toxic for eukaryotic cell lines, becomes active when coupled to a targeting moiety [34,35]. The structural model of this composite peptide is shown in Figure 12A. To determine the cytotoxicity of the fusion peptide, MM cells were treated with various concentrations before cell viability was evaluated by flow cytometry and propidium iodide (PI) staining. PI does not stain live cells, due to the presence of an intact plasma membrane. As shown in Figure 12B, the fusion peptide had no significant effect on normal PBMCs, whereas more than 70% of MM cells died following exposure to the MC-KLA fusion peptide. A summary of the cell viability data is shown in Figure 12C. Even at low peptide concentrations, a significant number of MM cells were killed. The IC50 values of the MC-KLA conjugate for U266 and CAG MM cells were 2.5 (±0.3) and 1.8 (±0.2) μM, respectively. Time-course analysis of cell viability using PI staining revealed a high percentage of cell death during the first 40 min of exposure to the fusion peptide (Figure 12D).

**Figure 13:**
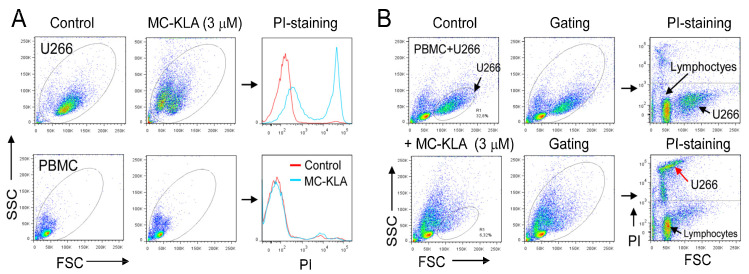
Effects of MC-KLA on MM cells under co-culture conditions with PBMCs. (**A**) U266 MM cells and PBMC were incubated with the MC-KLA fusion peptide (3 μM) separately for 60 min at 37 °C, stained with PI, and then analyzed by flow cytometry. Gated cells are indicated. The used PBMCs were depleted of monocytes by plastic adherence. In (**B**), we mixed U266 cells with PBMCs and then incubated the mixture with MC-KLA fusion peptide under the same conditions as in (**A**). Then the cells were incubated with PI and analyzed with flow cytometry to check for dead cells. The red arrow indicates the PI-stained U266 cells. Under these conditions, most U266 cells are killed but not PBMC.

To further confirm the lack of cytotoxicity on normal cells, PBMCs were mixed with U266 MM cells and then exposed to the MC-KLA fusion peptide for 60 min at 37 °C. As indicated by PI staining, exposure to MC-KLA fusion peptide did kill MM cells but not normal cells (Figure 13A,B). These data confirm that the fusion peptide, at the concentration used, specifically kills myeloma cells while having no significant effect on normal cells. Future work will focus on evaluating the cytotoxicity of the developed agents in animal models.

## 4. Discussion

Despite the significant advances in cancer immunotherapy, the majority of malignancies, including multiple myeloma, remain unresponsive to current immunotherapies [4]. In this study, we explored the use of phage display technology together with a whole-cell affinity selection approach to develop novel human scFv-Fc fusion proteins against MM cells. One of the selected scFvs was selected based on its strong binding to three MM cell lines and formatted into a human IgG1 derivative to make it available for cancer immunotherapy. The engineered scFv-Fc retained its binding specificity and recognized MM cell lines with high affinity. Additionally, it induced ADCC, an important anti-tumor mechanism of therapeutic antibodies. Competition experiments revealed that the scFv-Fc recognizes a super-sulfated heparan epitope that is preferentially expressed on MM cells. The binding is more likely mediated by the cell surface proteoglycan syndecan-1 since its knockdown in MM cells inhibited the scFv-Fc binding. The importance of the VH CDR2 and CDR3 in determining binding specificity was also demonstrated by designing a human heavy-chain-only nanobody and a peptide mimicking the nanobody paratope.

Notably, the malignant transformation of cells frequently induces dramatic alterations in the expression of cell surface molecules. In addition to aberrant glycosylations [35], genetic alterations can result in a change in the type, number, and arrangement of the cell surface receptors. To probe for such changes, we have used a semi-synthetic human scFv phage library. By contrast to purified protein targets, the affinity selection of phage libraries on live cells is more likely to enrich for scFvs that bind to cell surface receptors in their natural conformation (e.g., correct protein folding and interactions with neighboring proteins). However, affinity selection of the antibody libraries on live cells has proved to be challenging, mainly because of the tendency of phages to bind non-specifically to cells [19]. To reduce the presence of phages reacting with common receptors, the scFv library was successively pre-incubated with PBMCs and blood B cells. To reduce the unspecific binding of MM cells to the phage coat proteins, the cells were pre-incubated with the helper phage prior to the affinity selection. Under these experimental conditions, only three rounds of sequential affinity selection were required to isolate phage scFv clones with a strong binding capacity to MM cell lines. The reactivity with normal blood B cells and various B cell lymphomas originating from different stages of B cell development was negative or very weak.

The identification and characterization of tumor-specific markers remain a major goal in both understanding the cellular transformation observed in cancer and in developing therapeutic targets. Molecules that are tumor-specific and/or overexpressed in cancer are expected to have functional roles in tumor growth and metastasis. While scFvs selected from phage libraries can be used as carriers to deliver therapeutics without knowing their cell surface binding partners, receptor identification is critical for their clinical applications. Using polyanionic agents as competitors, the receptor of the MM1 scFv-Fc antibody was identified as a heavily sulfated heparan sulfate. Cell-surface heparan sulfate peptidoglycans (HSPGs) consist of two sub-classes, syndecans and glypicans, which are transmembrane and glycosylphosphatidylinositol-anchored peptidoglycans, respectively [25,26,36,37,38]. Syndecan-1 is an important member of the HSPG family and the dominant HSPG expressed by MM cells in humans [30]. The involvement of syndecan-1 in the MM1 scFv-Fc binding was demonstrated by the knockdown of its expression in MM cells and further confirmed by overexpressing it in HEK293T cells. Both results indicate that syndecan-1 HSPG is likely the MM1 binding receptor. In line with this observation, both the MM1 scFv-Fc receptor complexes and syndecan-1 co-localized on the surface of MM cells.

Heparin and heparan sulfate (HS) are made up of linear chains of repeating disaccharide units consisting of glucosamine and uronic acid [25,26]. Due to the complex synthesis and modifications, HS chains often show a high degree of heterogeneity with respect to chain length, disaccharide composition, and sulfation profile. Notably, it is the sulfation pattern/code of the glycan repeating units that generate the large structural and functional diversity. The glucosamine residues can either be *N*-sulfated or *N*-acetylated, both of which can undergo 6-*O*-sulfation, while the uronic acid residues can only be 2-*O*-sulfated [25,26]. The reported data indicate that the degree and nature of sulfonation determine the strength of the MM1 binding. In this respect, *N*-sulfated heparin displayed a remarkably stronger binding to the MM1 scFv-Fc than its *N*-acetylated counterpart. Since *N*-sulfation has been shown to be a dominant modification in MM cells, the binding of MM1 and its derivatives should be skewed towards MM cells as compared to other cell types and tissues. Although more work is needed, the epitope recognized by the scFv-Fc fusion protein is likely to contain at least a 2-*O*-sulfated uronic acid, and an N-sulfated glucosamine with the N-sulfation functioning as a potential switch, allowing the fusion protein to bind with very high affinity. It should be noted that, in addition to MM cells, the MM1 scFv-Fc antibody also bound tumor epithelial cells known to overexpress syndecan-1 [29,39,40], although the binding was 2–4-fold lower than that of MM cells. Moreover, at lower concentrations (1–50 ng/mL) the antibody demonstrated a significant binding to MM cells only [41].

Due to their small size, nanobodies and short peptides may address some challenges faced by cancer-targeted immunotherapy, such as the ability to penetrate into solid tumors. Unlike conventional antibodies, the heavy-chain antibodies of Camelidae contain a single variable domain (VHH) and two constant domains (CH2 and CH3) only [42]. It should be noted that the soluble human VH domain described here does not possess the feature amino acid changes present in the VHHs of camelid heavy chain antibodies, where four highly conserved and hydrophobic amino acids (V37, G44, L45, and W47) in VH are substituted by hydrophilic amino acids (F/Y37, E44, R45, and G47) in nanobodies [43]. Therefore, the increased number of positively charged amino acids within CDR2 and CDR3 may have contributed to the solubility of the expressed nanobody.

The development of targeting peptides directly from the amino acid sequence information of antibodies could lead to the development of a novel group of therapeutics. Strategies to develop peptides based on CDR loop sequences have been reported for some targets [44,45]. By modeling the antibody paratope sequences, we have identified a consensus CDR sequence that mimics the nanobody binding. Because of its ability to cross the plasma membrane of MM cells, the developed MC peptide can be used to deliver cargo that is specifically toxic to MM cells. In this respect, when the MC peptide was linked to a pro-apoptotic peptide, it killed MM cells. The fusion peptide did not affect the healthy donor PBMCs, highlighting a selective effect. Hence, the developed agent may represent a promising drug candidate for targeting MM cells.

Previous studies have shown that HSPGs play a crucial role in malignant cell growth by assembling signaling complexes and presenting growth factors to their cognate receptors [46,47,48]. Indeed, the sulfated HS side chains, bearing multiple negative charges, allow HSPGs to bind and interact with a broad variety of cytokines and growth factors in the tumor microenvironment [48]. Among the cellular events regulated by these HSPG-ligand interactions are cell proliferation, differentiation, adhesion, migration, angiogenesis, invasion, and metastasis [48]. In the bone marrow, syndecan-1 is highly expressed by the majority of malignant plasma cells, and its HS chains with highly modified domains would provide specific docking sites for a large number of bioactive molecules and growth factors such as hepatocyte growth factors and epidermal growth factors [38,39]. As with growth factor binding, interactions with the extracellular matrix appear to be facilitated through HS. The ligand binding sites seem to reside within distinct sulfated domains formed by chemical modifications of the HS disaccharide repeat. Hence, the inhibition of such interactions using high-affinity Fc-fusion antibodies such as MM1 could deprive cancer cells of multiple growth and angiogenic factors, which could be a valid therapeutic approach for treating MM patients. Moreover, the Fc region of the antibodies can activate Fc-dependent effector functions (ADCC, ADP, and CDC), boosting the antitumor effect.

Despite extensive research, antibodies targeting syndecan-1 are not yet approved for patients. However, several antibody-drug conjugates (ACDs) have been developed to deliver cytotoxic agents to tumor cells with elevated surface levels of syndecan-1. Among them, indatuximab ravtansine (BT062) is an ADC consisting of the anti-syndecan-1 chimeric monoclonal antibody, known as B-B4, and the microtubule-binding cytotoxic agent maytansinoid DM4 [49]. B-B4 antibody targets a region of the syndecan-1 protein, which is distally located from the cell membrane. As a monotherapy, BT062 showed a favorable safety profile but a very limited antitumor effect. In combination with some immunomodulatory drugs such as lenalidomide, BT062 improved overall survival in multiple myeloma patients who were refractory to lenalidomide [50]. Most of the developed antibodies recognize a similar protein sequence on the syndecan-1 ectodomain. The antibodies described in this study target the heparan surface on syndecan-1 core protein and could therefore show different therapeutic efficacy than B-B4. Of note, several antibodies targeting the same antigen (e.g., HER2, EGFR, CD20, and PD1 ligands) have been developed [14]. While these antibodies target the same antigen, their modes of action, pharmacokinetics, and clinical outcomes may differ from one another. Thus, the development of new candidate antibodies against antigens that have already been targeted by other approved antibodies should not be dismissed. Each type of antibody has different biophysical properties (e.g., affinity, solubility, and stability), and effector functions.

## 5. Conclusions

The affinity selection strategy described here led to the identification of a novel human scFv-Fc antibody that binds strongly to myeloma cells and activates NK cells for ADCC. Given the importance of syndecan-1 HSPG in several cancer types, including breast, colon, lung, and myeloma, the development of new HS-binding antibodies and antibody derivatives is important for therapy and for understanding the functional roles of HS sulfation in syndecan-1. Thus, our experimental strategy offers the opportunity to identify novel antibody derivatives against membrane molecules with desirable properties that may be used directly as a therapy or in other therapeutic forms such as CAR-T cells. The scFv-Fc-mimicking peptide was successfully utilized as a targeting moiety to deliver a pro-apoptotic peptide to MM cells and selectively kill these cells in vitro. Ligand-directed uptake of toxic drugs, as shown here, is intended to elevate potency and cell specificity.

## Figures and Tables

**Table 1:** Enrichment of phage binders during the affinity selection experiments.

Sequential Affinity Selection on Cancer Cell Lines	Cell Number	Input Number of Phages (TU)	Recovered Number of Phages (TU)	Enrichment over Previous Round *
CAG	1 × 10^7^	1 × 10^10^	2.0 × 10^3^	0
RPMI 8226	1 × 10^7^	1 × 10^10^	1.5 × 10^4^	5
U266	1 × 10^7^	1 × 10^10^	1.2 × 10^7^	800

* Refers to enrichment over the CAG-eluted phages, which are considered 0.

**Table 2:** Amino acid sequences of the heavy-chain and light-chain CDR2 and CDR3 regions.

Clone	VH CDR2	VH CDR3	VL CDR2	VL CDR3	Frequency *
MM1	AIRHPGLHTEY	AKGGRRFDY	RASRLQS	QQANSPPPT	21/30
MM10	TIRRQGGNTEY	AKSARVFDY	TASRLRS	QQWTAKPGT	2/30
MM12	AIRRPHLNTEY	AKGRRPRKFDY	RASHLQS	QQPNAPAPT	7/30

* The number of times each sequence occurred in the 30 sequenced MM phage-binding clones. Amino acid residues are indicated using a single-letter code. The CDRs are defined according to the international ImMunoGene Tics (TIMGT) numbering system(https://www.imgt.org, accessed on 20 December 2022).

## Data Availability

The data presented in this study are available on request from the corresponding author.

## Supplementary Materials

The following supporting information can be downloaded at: https://www.mdpi.com/article/10.3390/cancers15071934/s1, Figure S1: Binding of MM12 and MM10 scFv antibodies to U266 multiple myeloma cell line in the absence or presence of heparin sulfate or chondroitin surfate.

## Abbreviations

ADCC	Antibody-dependent cellular cytotoxicity
ADP	Antibody-dependent phagocytosis
CDC	Complement-dependent cytotoxicity
CDR	Complementarity-determining regions
HS	Heparan sulfate
HSPGs	Heparan sulfate peptidoglycans
MM	Multiple myeloma
PBMCs	Peripheral blood mononuclear cells
PE	Phycoerythrin
PEG	Polyethylene glycol
PI	Propidium iodide
scFv	Single-chain variable fragment
siRNA	Small interfering RNA
V_H_	Heavy variable chain
V_L_	Light variable chain
