# Supplementary material for: Antibody Surface Profiling Identifies Glycoforms in Multiple Myeloma as Targets for Immunotherapy: From Antibody Derivatives to Mimetic Peptides for Killing Tumor Cells
Source: Cancers (Basel). 2023 Mar 23;15(7):1934. doi: 10.3390/cancers15071934 (PMC10093763; doi:10.3390/cancers15071934)
Supplement: Supplementary file 1 [file cancers-15-01934-s001.zip › cancers-2274149-supplementary.pdf]

Mouldy Sioud and Anniken Olberg

Antibody Surface Profiling Identifies Glycoforms in Multiple Myeloma as Target for Immunotherapy: From Antibody Derivatives to Mimetic Peptides for Killing Tumor Cells

Cancers 2023, 15.

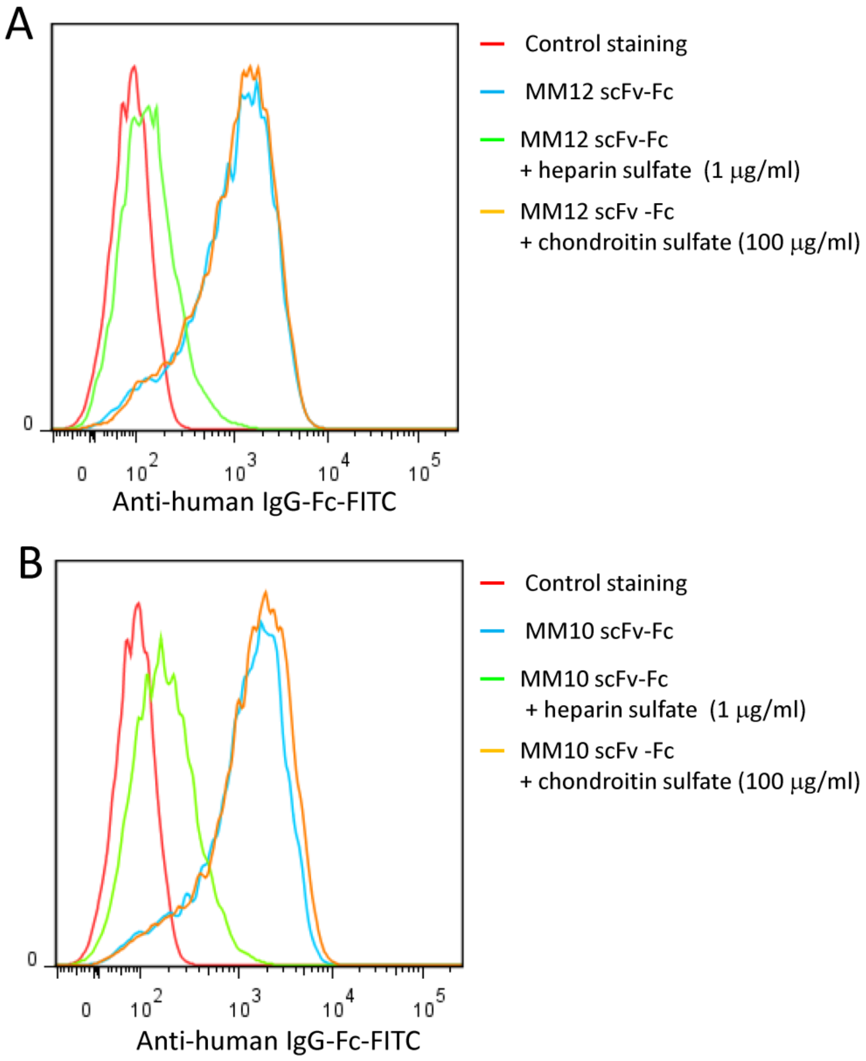

Supplementary Figure S1. Binding of MM12 and MM10 scFV-Fc antibodies to U266 multiple myeloma cell line in the absence or presence of heparin sulfate or chondroitin sulfate. The antibodies were incubated with the indicated competitors for 20 min at room temperature prior to the addition of U266 MM cells and FACS analysis as described in Materials and Methods.
